# Metabolic obesity phenotypes and the risk of cancer: a prospective study of the Kailuan cohort

**DOI:** 10.3389/fendo.2024.1333488

**Published:** 2024-10-16

**Authors:** Xin Zheng, Yiming Wang, Yue Chen, Tong Liu, Chenan Liu, Shiqi Lin, Hailun Xie, Xiangming Ma, Ziwen Wang, Jinyu Shi, Heyang Zhang, Ming Yang, Xiaoyue Liu, Li Deng, Qingsong Zhang, Hanping Shi

**Affiliations:** ^1^ Department of Gastrointestinal Surgery, Beijing Shijitan Hospital, Capital Medical University, Beijing, China; ^2^ Department of Clinical Nutrition, Beijing Shijitan Hospital, Capital Medical University, Beijing, China; ^3^ Beijing International Science and Technology Cooperation Base for Cancer Metabolism and Nutrition, Beijing, China; ^4^ Key Laboratory of Cancer Food for Special Medical Purposes (FSMP) for State Market Regulation, Beijing, China; ^5^ Department of Hepatological Surgery, Kailuan General Hospital, Tangshan, China; ^6^ The Second Affiliated Hospital and Yuying Children’s Hospital of Wenzhou Medical University, Wenzhou, China; ^7^ Department of General Surgery, Kailuan General Hospital, Tangshan, China

**Keywords:** metabolic health status, single obesity, abdominal obesity, cancer morbidity, all-cause mortality

## Abstract

**Background:**

Obesity is as an important risk factor for chronic diseases. Metabolically healthy obesity (MHO) is considered a benign state. The association between metabolic health and obesity categories and cancer risk remains unclear. This study aimed to investigate the relationship between metabolic health status combined with obesity phenotypes and the risk of cancer.

**Methods:**

Data from 91,834 participants in the Kailuan cohort were analyzed, excluding individuals with a body mass index (BMI) < 18.5 kg/m² and those with a history of cancer. Obesity phenotypes were classified based on BMI and waist circumference (WC) combined with metabolic health status, resulting in six phenotypes. Cox proportional hazard regression models were used to assess the association between metabolic health and obesity phenotypes with cancer risk and all-cause mortality.

**Results:**

The prevalence of metabolically healthy obesity and metabolically unhealthy obesity defined by BMI was 6.86% and 12.18%, while that defined by WC was 20.79% and 25.76%, respectively. Compared to metabolically healthy participants, individuals with an unhealthy metabolic status had a significantly higher risk of cancer (HR, 1.09; 95% CI, 1.03–1.15; p=0.004). The hazard ratios for cancer were 1.19, 1.23, 1.20, and 1.55 for individuals with one, two, three, and four metabolic disorders, respectively. Among those classified as metabolically unhealthy, both overweight and obesity were associated with a protective effect on cancer risk (HR, 0.88; 95% CI, 0.80–0.96; p=0.006 for overweight; HR, 0.87; 95% CI, 0.78–0.97; p=0.010 for obesity). However, abdominal obesity significantly increased cancer risk in both metabolically healthy and unhealthy participants. In subgroup analysis, simple obesity showed a protective trend against cancer in those with respiratory cancers, while abdominal obesity consistently posed a risk for various cancer types.

**Conclusion:**

Metabolically unhealthy status and abdominal obesity are risk factors for cancer and all-cause mortality, whereas simple obesity offers protective effects against cancer and all-cause mortality in metabolically unhealthy individuals. These findings suggest that maintaining metabolic health and reducing the metabolic risks associated with abdominal obesity should be key targets for cancer prevention.

## 1 Introduction

The global cancer burden continues to increase (1). Although cancer mortality and morbidity rates vary between countries and regions, cancer remains a major cause of mortality worldwide, posing a significant public health challenge (2, 3). In the context of a fast-growing and aging population, cancer remains a major impediment to life expectancy, and major high-risk cancers have not yet shown significant downward trends (1). In 2019, more than 10 million people died of cancer and approximately 23 million people had cancer, about twice as many as in 1990 (2). However, since 1990, the age-standardized mortality rate has shown a decreasing trend, while the age-standardized incidence rate has shown an increasing trend (2). Therefore, more cancer prevention strategies targeting major risk factors should be proposed and actively implemented to reduce the global cancer burden.

In recent years, the composition of diets have changed dramatically, with a much higher proportion of carbohydrate intake, leading to obesity becoming a non-negligible problem in developed and developing countries (4). Previous studies suggested that obesity contributes to the development of 13 types of cancer, and the concept of obesity-related tumors (including endometrial, breast, esophageal, colorectal, gastric, liver, kidney cancers) has been proposed (5). Based on body mass index (BMI) and waist circumference (WC), obesity can be divided into simple and abdominal obesity. Abdominal obesity is considered a sign of increased ectopic fat (around the liver, heart, skeletal muscle and pancreas) and people with abdominal obesity are more prone to metabolic and cardiovascular diseases (6). The high prevalence of obesity worldwide has also raised concerns about the metabolic health. Metabolic health is defined as the absence of metabolic syndrome, diabetes, hypertension, or dyslipidemia (7). Notably, not all individuals with normal weight have disease-free characteristics and a healthy metabolic phenotype, and even individuals who are overweight or obese have heterogeneous metabolic health (8). It is estimated that approximately 20% of normal weight adults exhibit metabolically unhealthy status and have increased risk factors associated with obesity, such as elevated blood pressure, insulin resistance, dyslipidemia, and cardiovascular events (7). In addition, people with metabolically healthy obesity (MHO) have normal blood lipids, insulin sensitivity, and plasma glucose despite an elevated BMI or WC, and they are less likely to develop cardiovascular disease than individuals with metabolically unhealthy obesity (MUO) (9, 10).

MUO is generally considered to increase the risk of cardiovascular disease and cancer. A previous meta-analysis focused on the effect of obesity on the risk of cancer in metabolically healthy people and suggested a significantly increased risk of cancer in MHO compared to metabolically healthy normal weight (MHNW) individuals (11). Another meta-analysis focused on the effect of metabolic health status on the risk of cancer in obese people and showed a decreased risk of cancer in MHO compared to MUO, regardless of population heterogeneity (12). Our study is one of the few to explore the association between metabolic health status combined with BMI and WC categories and the risk of cancer.

## 2 Materials and methods

### 2.1 Study population

**Figure 1:**
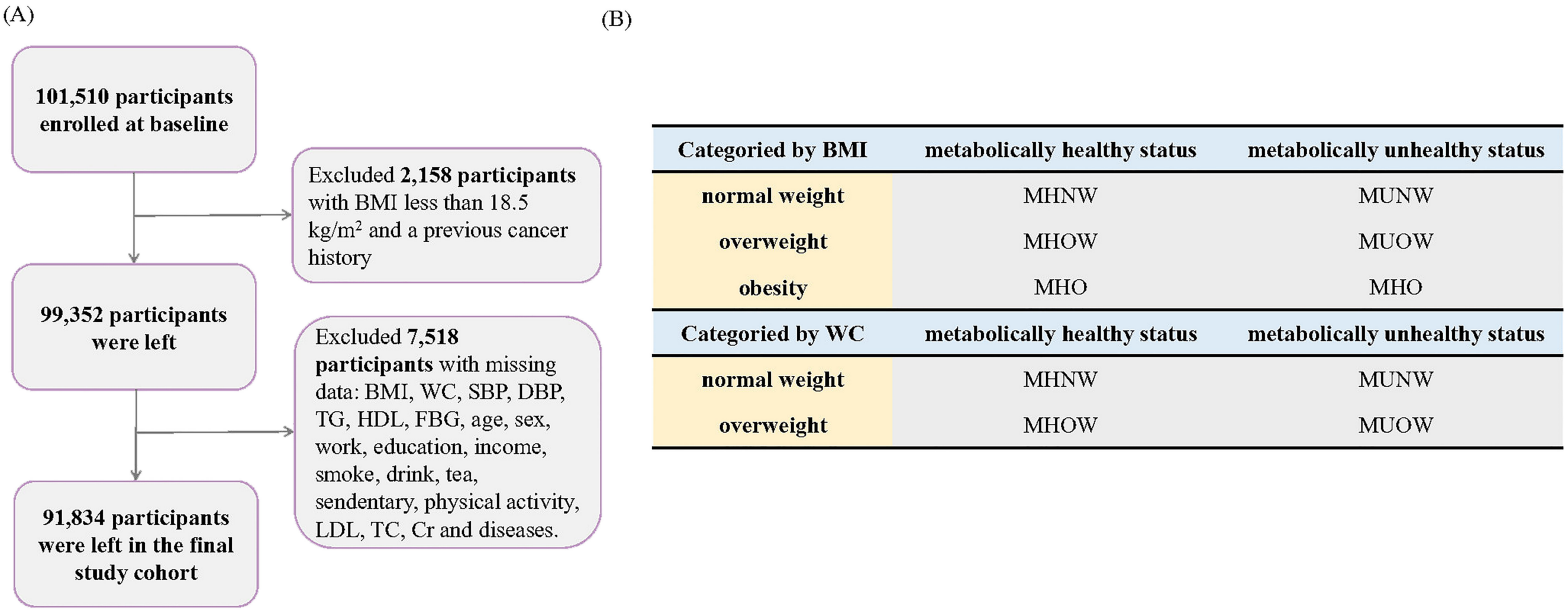
Flow chart. **(A)** Participant Selection and Enrollment Flowchart, **(B)** Classification of Participants by BMI and Waist Circumference (WC).

The Kailuan study is a prospective cohort study conducted in Tangshan, China (13). The specific details and procedural plan of the study have been reported previously (14). Briefly, starting in 2006, participants received a thorough checkup and subsequent updates of their health status every two years, including questionnaires, routine physical examinations, and blood and urine tests. In the present study, we included 101,510 participants enrolled at baseline, excluding those with BMI less than 18.5 kg/m^2^ or previous cancer history, leaving 99,352 participants. Next, we excluded 2,511 participants with missing values for the exposure variables (metabolic health status and obesity status: BMI, WC, systolic blood pressure, diastolic blood pressure, triglycerides, high-density lipoprotein [HDL], and fasting blood glucose [FBG]) and 5,007 participants with missing values for covariates, leaving 91,834 participants for the association analysis (**Figure 1A**).

### 2.2 Exposure factors

BMI was calculated based on the following formula: BMI = weight/height (kg/m^2^). Based on the recommendations of the China Obesity Working Group, the obesity categories were defined based on BMI and WC criteria (15). Based on the BMI criteria, participants were classified as normal-weight (18.5 kg/m2 < BMI < 24 kg/m2), simple overweight (24 kg/m^2^ < BMI < 28 kg/m^2^) and simple obese (BMI > 28 kg/m^2^). Based on the WC criteria, participants were classified as normal weight (WC < 85 cm for men and < 80 cm for women) and abdominally obese (WC ≥ 85 cm for men and ≥ 80 cm for women). We utilized standardized operating protocols to measure four Adult Treatment Panel-III (ATP-III) components in order to define metabolic syndrome (16). Participants with two or more of the four criteria were considered to be metabolically unhealthy: (1) systolic blood pressure > 130 mmHg or diastolic blood pressure > 85 mmHg, use of antihypertensive medications, or self-reported physician-diagnosed hypertension; (2) triglycerides ≥ 1.7 mmol/L or use of antihyperlipidemic drugs; (3) FBG ≥ 5.6 mmol/L, use of antihyperglycemic drugs, or self-reported physician-diagnosed diabetes mellitus; or (4) HDL ≤ 1.04 mmol/L and ≤ 1.29 mmol/L in men and women, respectively. According the BMI categories (normal-weight, simple overweight, and simple obesity), participants were classified into six phenotypes: MHNW, metabolically healthy overweight (MHOW), MHO, metabolically unhealthy normal-weight (MUNW), metabolically unhealthy overweight (MUOW), and MUO. According to the WC categories (normal weight and abdominal obesity), participants were classified into four phenotypes: MHNW, MHO, MUNW, and MUO (**Figure 1B**) (17).

### 2.3 Covariates

Participants were interviewed face-to-face and clinically examined by medical professionals to collect socio-demographic data (sex, age, income, education, and occupation); lifestyle data (sedentary, physical activity amount, smoking status, alcohol consumption, and tea consumption); hematology test results (serum creatinine); and disease information (fatty liver, hepatitis B, cirrhosis, gallstones, and gallbladder polyps). Smoking and drinking status were categorized as never, former, or current. The physical activity levels were categorized as never, occasionally, or frequently. Education was categorized as middle school or below, high school or above. Income was categorized as < $600/month, $600–800/month, $800–1000/month, or ≥ $1000/month. Blood samples were collected after fasting for 8 to 12 hours, then serum creatinine and serum lipid levels were measured by an autoanalyzer.

### 2.4 Outcome assessment

Cancer incidence events during follow-up were confirmed by interviewing participants and examining hospital diagnostic records. Cancer types were identified using International Classification of Diseases-10 (ICD-10) codes. In this study, we analyzed the association between metabolic health status combined with obesity status and the risk of cancer, and refined them into the following categories, including respiratory cancer (C34–C39), digestive cancer (C15–C26), and other systemic cancers (all codes begin with C).

### 2.5 Statistical analysis

The baseline table was constructed according to metabolic health scores, with continuous variables expressed as median [interquartile range (IQR)] (skewed distribution) and mean [standard deviation (SD)] (normal distribution). Categorical variables were expressed as numbers (percentages). Hazard risk (HRs) and 95% confidence intervals (CIs) were calculated using the Cox proportional risk regression models (this study met the proportional risk assumption). Model 1 adjusted for sex and age; model 2 additionally adjusted for education, occupation, income, sedentary lifestyle, physical activity, smoking status, alcohol status, and tea consumption; and model 3 additionally adjusted for serum creatinine, hepatitis B, cirrhosis, gallstones, gallbladder polyps, and fatty liver. First, we investigated the association between metabolic health scores and the risk of cancer. Second, we assessed the association between MUNW, MHOW, MUOW, MHO, and MUO with the risk of cancer using MHNW as a dummy variable. Then, we further investigated the following two effects: (1) The effect of obesity categories on the risk of cancer among different metabolically healthy status and (2) the effect of metabolic health status on the risk of cancer among different obesity categories. We additionally analyzed the association between metabolic health status combined with obesity categories and all-cause mortality. Finally, two sensitivity analyses were performed to clarify the robustness of the results: (1) exclusion of participants with cancers occurring within 1 year to exclude the possibility of reverse causation; and (2) additional adjustment for low-density lipoprotein (LDL) and total cholesterol (TC). All statistical analyses were performed using R software version 4.2.0 (R Foundation for Statistical Computing, Vienna, Austria). A two-tailed p < 0.05 was considered statistically significant.

## 3 Results

### 3.1 Baseline characteristics

Differences between groups with different metabolic health scores were compared, and the results are presented in **Table 1**. Of the 91,834 participants included in the study, 50,054 participants were metabolically healthy. As metabolic health scores increased, age and inflammation progressively increased, metabolic health status progressively became worse, and cancer incidence and all-cause mortality progressively increased. Metabolically unhealthy individuals were more likely to be men, have a lower education level, be current smokers, and have a higher BMI and WC. In addition, the metabolically unhealthy group included more individuals with fatty liver and gallstones.

**Table 1:** Baseline characteristics.

Characteristics	level	Overall	Score 0	Score 1	Score 2	Score 3	Score 4	p
**n**		91834	18780	31274	27553	12898	1329	
**Age (mean (SD))**		51.43 (12.35)	46.30 (12.43)	51.72 (12.53)	52.99 (11.82)	54.39 (10.81)	55.95 (9.98)	<0.001
**Sex (%)**	Woman	18323 (19.95)	5237 (27.89)	5749 (18.38)	4744 (17.22)	2233 (17.31)	360 (27.09)	<0.001
	Man	73511 (80.05)	13543 (72.11)	25525 (81.62)	22809 (82.78)	10665 (82.69)	969 (72.91)	
**Occupation (%)**	Brain	6949 (7.57)	1763 (9.39)	2195 (7.02)	1904 (6.91)	956 (7.41)	131 (9.86)	<0.001
	Physical	84885 (92.43)	17017 (90.61)	29079 (92.98)	25649 (93.09)	11942 (92.59)	1198 (90.14)	
**Education (%)**	Middle school and above	73780 (80.34)	13474 (71.75)	25487 (81.50)	22909 (83.15)	10826 (83.94)	1084 (81.57)	<0.001
	High school and above	18054 (19.66)	5306 (28.25)	5787 (18.50)	4644 (16.85)	2072 (16.06)	245 (18.43)	
**Income (%)**	<600	26625 (28.99)	5219 (27.79)	8737 (27.94)	8199 (29.76)	4040 (31.32)	430 (32.36)	<0.001
	600-800	52109 (56.74)	10473 (55.77)	18227 (58.28)	15674 (56.89)	7022 (54.44)	713 (53.65)	
	800-1000	7016 (7.64)	1533 (8.16)	2390 (7.64)	1968 (7.14)	1016 (7.88)	109 (8.20)	
	>1000	6084 (6.62)	1555 (8.28)	1920 (6.14)	1712 (6.21)	820 (6.36)	77 (5.79)	
**Smoking status (%)**	Never	54868 (59.75)	11539 (61.44)	18941 (60.56)	16091 (58.40)	7435 (57.64)	862 (64.86)	<0.001
	Former	5255 (5.72)	771 (4.11)	1701 (5.44)	1737 (6.30)	959 (7.44)	87 (6.55)	
	Current	31711 (34.53)	6470 (34.45)	10632 (34.00)	9725 (35.30)	4504 (34.92)	380 (28.59)	
**Alcohol consumption (%)**	Never	54043 (58.85)	11080 (59.00)	18764 (60.00)	15925 (57.80)	7412 (57.47)	862 (64.86)	<0.001
	Former	3559 (3.88)	509 (2.71)	1122 (3.59)	1225 (4.45)	636 (4.93)	67 (5.04)	
	Current	34232 (37.28)	7191 (38.29)	11388 (36.41)	10403 (37.76)	4850 (37.60)	400 (30.10)	
**Tea consumption (%)**	No	73009 (79.50)	14888 (79.28)	25222 (80.65)	21892 (79.45)	9970 (77.30)	1037 (78.03)	<0.001
	Yes	18825 (20.50)	3892 (20.72)	6052 (19.35)	5661 (20.55)	2928 (22.70)	292 (21.97)	
**Sedentary (%)**	<4h	68796 (74.91)	13508 (71.93)	23934 (76.53)	20804 (75.51)	9601 (74.44)	949 (71.41)	<0.001
	4h-8h	20121 (21.91)	4588 (24.43)	6362 (20.34)	5961 (21.63)	2865 (22.21)	345 (25.96)	
	≥8h	2917 (3.18)	684 (3.64)	978 (3.13)	788 (2.86)	432 (3.35)	35 (2.63)	
**Physical activity (%)**	Never	8046 (8.76)	1625 (8.65)	2663 (8.52)	2488 (9.03)	1154 (8.95)	116 (8.73)	<0.001
	Occasionally	69474 (75.65)	14863 (79.14)	23959 (76.61)	20609 (74.80)	9248 (71.70)	795 (59.82)	
	Frequently	14314 (15.59)	2292 (12.20)	4652 (14.87)	4456 (16.17)	2496 (19.35)	418 (31.45)	
**BMI (mean (SD))**		25.20 (3.38)	23.66 (3.00)	24.87 (3.17)	25.85 (3.36)	26.63 (3.39)	27.05 (3.49)	<0.001
**WC (mean (SD))**		87.17 (9.80)	82.84 (9.43)	86.43 (9.42)	88.92 (9.40)	91.08 (9.49)	91.68 (9.16)	<0.001
**SBP (mean (SD))**		131.26 (21.03)	112.92 (9.45)	130.48 (19.92)	137.76 (20.13)	144.47 (19.54)	145.45 (17.87)	<0.001
**DBP (mean (SD))**		83.73 (11.76)	74.48 (6.71)	83.30 (11.09)	87.12 (11.50)	90.31 (11.35)	90.46 (10.80)	<0.001
**TG (median [IQR])**		1.28 [0.91, 1.94]	0.94 [0.70, 1.21]	1.16 [0.87, 1.54]	1.70 [1.11, 2.46]	2.09 [1.46, 3.05]	1.85 [1.24, 2.85]	<0.001
**HDL (median [IQR])**		1.50 [1.28, 1.76]	1.55 [1.36, 1.77]	1.54 [1.33, 1.78]	1.49 [1.26, 1.77]	1.40 [1.11, 1.71]	0.96 [0.87, 1.02]	<0.001
**LDL (median [IQR])**		2.35 [1.85, 2.84]	2.17 [1.71, 2.64]	2.36 [1.88, 2.80]	2.40 [1.92, 2.90]	2.48 [1.92, 3.04]	2.40 [1.75, 3.04]	<0.001
**FBG (median [IQR])**		5.12 [4.68, 5.73]	4.84 [4.50, 5.15]	5.00 [4.60, 5.39]	5.31 [4.78, 5.99]	6.10 [5.63, 7.18]	6.29 [5.84, 7.70]	<0.001
**TC (median [IQR])**		4.94 [4.29, 5.60]	4.69 [4.16, 5.20]	4.89 [4.30, 5.47]	5.09 [4.37, 5.85]	5.26 [4.43, 6.20]	4.68 [4.04, 5.47]	<0.001
**Cr (median [IQR])**		89.40 [76.60, 103.80]	86.90 [75.00, 99.00]	90.00 [77.00, 104.10]	90.90 [77.00, 105.60]	91.00 [77.00, 106.00]	89.00 [77.00, 101.00]	<0.001
**Fatty liver (%)**	No	62217 (67.75)	16263 (86.60)	23269 (74.40)	16258 (59.01)	5869 (45.50)	558 (41.99)	<0.001
	Yes	29617 (32.25)	2517 (13.40)	8005 (25.60)	11295 (40.99)	7029 (54.50)	771 (58.01)	
**Cancer (%)**	No	86431 (94.12)	17886 (95.24)	29383 (93.95)	25828 (93.74)	12111 (93.90)	1223 (92.02)	<0.001
	Yes	5403 (5.88)	894 (4.76)	1891 (6.05)	1725 (6.26)	787 (6.10)	106 (7.98)	
**Death (%)**	No	78353 (85.32)	17449 (92.91)	26822 (85.76)	22785 (82.70)	10254 (79.50)	1043 (78.48)	<0.001
	Yes	13481 (14.68)	1331 (7.09)	4452 (14.24)	4768 (17.30)	2644 (20.50)	286 (21.52)	
**Hepatitis B (%)**	No	89306 (97.25)	18159 (96.69)	30394 (97.19)	26846 (97.43)	12608 (97.75)	1299 (97.74)	<0.001
	Yes	2528 (2.75)	621 (3.31)	880 (2.81)	707 (2.57)	290 (2.25)	30 (2.26)	
**Liver cirrhosis (%)**	No	91673 (99.82)	18740 (99.79)	31206 (99.78)	27514 (99.86)	12886 (99.91)	1327 (99.85)	0.021
	Yes	161 (0.18)	40 (0.21)	68 (0.22)	39 (0.14)	12 (0.09)	2 (0.15)	
**Gallstones (%)**	No	89836 (97.82)	18467 (98.33)	30650 (98.00)	26894 (97.61)	12526 (97.12)	1299 (97.74)	<0.001
	Yes	1998 (2.18)	313 (1.67)	624 (2.00)	659 (2.39)	372 (2.88)	30 (2.26)	
**Biliary polyps (%)**	No	91079 (99.18)	18591 (98.99)	31014 (99.17)	27351 (99.27)	12804 (99.27)	1319 (99.25)	0.017
	Yes	755 (0.82)	189 (1.01)	260 (0.83)	202 (0.73)	94 (0.73)	10 (0.75)	

Continuous variables are presented as mean (SD) or median [IQR], and categorical variables are presented as numbers (percentages).

BMI, body mass index; WC, waist circumference; SBP, systolic blood pressure; DBP, diastolic blood pressure; TG, triglycerides; HDL, high density cholesterol; LDL, low density cholesterol; TC, total cholesterol; FBG, fasting blood glucose; CRP, C-reactive protein; Cr, creatinine; SD, standard deviation; IQR, inter-quartile range.

### 3.2 Association between metabolic health and the risk of cancer

We first investigated the association between metabolic health status and the risk of cancer, and the results are displayed in **Table 2**. After fully adjusting for covariates, participants with a metabolically unhealthy status had a 9% increased risk compared to those with metabolically healthy status (HR, 1.09; 95% CI, 1.03–1.15; p =0.004). We next investigated the association between metabolic health scores and the risk of cancer. The risk of cancer was increased by 19%, 23%, 20%, and 55% in the presence of one, two, three, and four metabolic disorders, respectively. It was suggested that the risk of cancer was already increased when one metabolic index was disordered, and the risk was significantly increased when four metabolic indexes were disordered simultaneously.

**Table 2:** The association between metabolic healthy score and the risk of cancer.

Group	Type	Cancer/total paticipants	Model 1	p	Model 2	p	Model 3	p
**Metabolic type**	**Metabolic healthy status**	2785/50054	ref.	ref.	ref.	ref.	ref.	ref.
	**Metabolic unhealthy status**	2618/41780	1.15 (1.09,1.21)	<0.001	1.10 (1.04,1.16)	<0.001	1.09 (1.03,1.15)	0.004
**Score**	**Score0**	894/18780	ref.	ref.	ref.	ref.	ref.	ref.
	**Score1**	1891/31274	1.27 (1.17,1.37)	<0.001	1.20 (1.11,1.30)	<0.001	1.19 (1.11,1.31)	<0.001
	**Score2**	1725/27553	1.33 (1.23,1.45)	<0.001	1.24 (1.15,1.35)	<0.001	1.23 (1.14,1.34)	<0.001
	**Score3**	787/12898	1.32 (1.20,1.45)	<0.001	1.22 (1.10,1.34)	<0.001	1.20 (1.10,1.32)	<0.001
	**Score4**	106/1329	1.77 (1.45,2.17)	<0.001	1.59 (1.30,1.94)	<0.001	1.55 (1.27,1.91)	<0.001
**p for trend**			<0.001		<0.001			<0.001

Model 1: adjusted for age and sex; Model 2: adjusted for Model 1, education, work, income, sedentary, physical activity, smoke, salt, drink, tea; Model 3, adjusted for Model2, hepatitis B, liver cirrhosis, gallstones, biliary polyps, fatty liver and creatinine.

MH, metabolic healthy; MU, metabolic unhealthy.

### 3.3 Association between metabolically healthy status combined with obesity categories (defined by BMI or WC) and the risk of cancer

The previous results suggested that a metabolically unhealthy status was a risk factor, and thus, we explored the association between metabolic health status combined with obesity categories and the risk of cancer. We first defined obesity based on BMI and using MHNW as a dummy variable found a 15% increased risk of cancer in MUNW individuals and a 9% decreased risk of cancer in MHOW individuals (**Supplementary Table 1**). Then, we divided the participants into metabolically healthy and metabolically unhealthy subgroups to explore the association between obesity categories and the risk of cancer. We next divided the participants into normal-weight, overweight, and obesity subgroups to explore the association between metabolic health status and the risk of cancer (**Table 3**). Interestingly, among metabolically unhealthy participants, overweight and obesity showed a protective trend on the risk of cancer, reducing the risk by 12% (HR, 0.88; 95% CI, 0.80–0.96; p =0.006) and 13% (HR, 0.87; 95% CI, 0.78–0.97; p =0.010), respectively. In the normal-weight and simple overweight subgroups, metabolically unhealthy status increased the risk of cancer by 15% (HR, 1.15; 95% CI, 1.05–1.26; p =0.002) and 10% (HR, 1.10; 95% CI, 1.02–1.20; p =0.020), respectively. However, in the obesity subgroup, the association between metabolically unhealthy status and the risk of cancer was not significant (HR, 0.98; 95% CI, 0.87–1.12; p =0.810), suggesting that simple obesity may act as a protective factor that partially resisted the increased risk by metabolically unhealthy status. In addition, we defined obesity based on WC and used MHNW as a dummy variable. The results suggested that MUNW, MHOW, and MUOW increased the risk of cancer by 11%, 16%, and 21%, respectively (p for trend<0.001) (**Supplementary Table 2**). Using the above subgroups, we further performed the same analyses (**Table 4**). We found that abdominal obesity increased the risk of cancer in both the metabolically healthy and unhealthy subgroups. In the normal-weight participants, metabolically unhealthy status also increased the risk of cancer. Furthermore, although the association between metabolically unhealthy status and the risk of cancer was not significant in the participants with abdominal obesity (HR, 1.04; 95% CI, 0.96–1.12; p =0.0342), there was still a hazardous trend. Finally, we explored the association between metabolic health status combined with obesity categories with the risk of different cancers (**Supplementary Figure 1**). We found that the protective effect of simple obesity on the risk of cancer was mainly present in individuals with respiratory cancer. However, abdominal obesity was a risk factor for both metabolically healthy and unhealthy participants (although not statistically significant).

**Table 3:** The association between metabolic healthy status combined with obesity categories (defined on BMI) and the risk of cancer.

Group	Type	Cancer/total participants	Model 1	p	Model 2	p	Model 3	p
Metabolic healthy	MHNW	1355/23988	ref.	ref.	ref.	ref.	ref.	ref.
	MHOW	1052/19769	0.94 (0.86,1.02)	0.113	0.94 (0.86,1.02)	0.116	0.91 (0.84,0.99)	0.031
	MHO	378/6297	1.07 (0.95,1.20)	0.266	1.08 (0.96,1.21)	0.190	1.01 (0.90,1.14)	0.864
**p for trend**				0.898		0.797		0.503
Metabolic unhealthy	MUNW	756/11211	ref.	ref.	ref.	ref.	ref.	ref.
	MUOW	1183/19382	0.90 (0.82,0.99)	0.025	0.91 (0.83,1.00)	0.043	0.88 (0.80,0.96)	0.006
	MUO	679/11187	0.90 (0.81,1.00)	0.055	0.93 (0.84,1.03)	0.155	0.87 (0.78,0.97)	0.010
**p for trend**				0.043		0.115		0.008
Normal weight	MHNW	1355/23988	ref.	ref.	ref.	ref.	ref.	ref.
	MUNW	756/11211	1.22 (1.11,1.33)	<0.001	1.15 (1.06,1.26)	0.002	1.15 (1.05,1.26)	0.002
Overweight	MHOW	1052/19769	ref.	ref.	ref.	ref.	ref.	ref.
	MUOW	1183/19382	1.17 (1.08,1.27)	<0.001	1.12 (1.03,1.22)	0.007	1.10 (1.02,1.20)	0.020
Obesity	MHO	378/6297	ref.	ref.	ref.	ref.	ref.	ref.
	MUO	679/11187	1.03 (0.91,1.17)	0.641	0.99 (0.87,1.13)	0.901	0.98 (0.87,1.12)	0.810

Model 1: adjusted for age and sex; Model 2: adjusted for Model 1, education, work, income, sedentary, physical activity, smoke, salt, drink, tea; Model 3, adjusted for Model2, hepatitis B, liver cirrhosis, gallstones, biliary polyps, fatty liver and creatinine.

MHNW, metabolic healthy normal weight; MUNW, metabolic unhealthy normal weight; MHOW, metabolic healthy overweight; MUOW, metabolic unhealthy overweight; MHO, metabolic healthy obesity; MUO, metabolic unhealthy obesity; BMI, body mass index.

**Table 4:** The association between metabolic healthy status combined with obesity categories (defined on WC) and the risk of cancer.

Group	Type	Cancer/total participants	Model 1	p	Model 2	p	Model 3	p
Metabolic healthy	MHNW	1595/30960	ref.	ref.	ref.	ref.	ref.	ref.
	MHO	1190/19094	1.22 (1.13,1.32)	<0.001	1.19 (1.10,1.28)	<0.001	1.16 (1.08,1.26)	<0.001
Metabolic unhealthy	MUNW	1067/18124	ref.	ref.	ref.	ref.	ref.	ref.
	MUO	1551/23656	1.13 (1.04,1.22)	0.002	1.12 (1.03,1.21)	0.005	1.09 (1.01,1.18)	0.035
Normal weight	MHNW	1595/30960	ref.	ref.	ref.	ref.	ref.	ref.
	MUNW	1067/18124	1.16 (1.07,1.25)	<0.001	1.11 (1.02,1.19)	0.012	1.11 (1.02,1.20)	0.012
Obesity	MHO	1190/19094	ref.	ref.	ref.	ref.	ref.	ref.
	MUO	1551/23656	1.07 (0.99,1.16)	0.076	1.04 (0.97,1.12)	0.288	1.04 (0.96,1.12)	0.342

Model 1: adjusted for age and sex; Model 2: adjusted for Model 1, education, work, income, sedentary, physical activity, smoke, salt, drink, tea; Model 3, adjusted for Model2, hepatitis B, liver cirrhosis, gallstones, biliary polyps, fatty liver and creatinine.

MHNW, metabolic healthy normal weight; MUNW, metabolic unhealthy normal weight; MHO, metabolic healthy obesity (central); MUO, metabolic unhealthy obesity (central); WC, waist circumference.

### 3.4 Association between metabolic health status combined with obesity categories (defined by BMI or WC) and all-cause mortality

The present study also addressed the association between metabolic health status combined with obesity categories and all-cause mortality (**Supplementary Table 3**, **Supplementary Figure 2**). First, metabolically unhealthy participants had a 44% increased risk of all-cause mortality, and the effect was consistent among normal-weight, overweight, and obese participants. In addition, participants had a progressively higher risk of mortality as metabolic health scores increased. Notably, simple overweight showed a protective trend against all-cause mortality in the metabolically healthy participants. In the metabolically unhealthy participants, simple overweight and obesity reduced the risk of all-cause mortality by 10% and 8%, respectively. However, abdominal obesity and metabolically unhealthy status were risk factors for all-cause mortality.

### 3.5 Sensitivity analysis

We performed two sensitivity analyses to assess the robustness of the results (**Supplementary Figure 3**). Metabolically unhealthy status increased the risk of cancer in both normal-weight and simple obesity participants, and among the metabolically unhealthy participants, simple obesity showed a protective trend for the risk of cancer. After excluding those who developed cancers within 1 year, abdominal obesity increased the risk of cancer in the metabolically healthy participants by 17% and metabolically unhealthy status increased the risk of cancer in the normal-weight participants by 11%. After additional adjustment for LDL and TC, abdominal obesity increased the risk of cancer in both metabolically healthy and unhealthy participants.

## 4 Discussion

This study examined the association between metabolic health status combined with obesity categories and cancer morbidity. We found that metabolically unhealthy status and abdominal obesity were risk factors for an increased risk of cancer and all-cause mortality. In addition, simple obesity was a protective factor against the risk of cancer and all-cause mortality in participants with a metabolically unhealthy status.

There was a significant association between overweight and obesity and metabolic-related diseases, involving multiple factors and complex pathogenesis (18). Genetic and epigenetic alterations (including DNA methylation and histone modifications) in the organism may affect energy metabolism, lipid metabolism and other metabolic pathways (19, 20). In the visceral fat of obese individuals, immune cells tend to exhibit a pro-inflammatory profile, and this becomes an important trigger for the development of obesity-related comorbidities. Multiple immune cell types, such as regulatory T cells, adipose tissue macrophages, dendritic cells and B cells are involved in maintaining adipose tissue homeostasis (21, 22).The adipose tissue consists mainly of white adipose tissue, which stores energy, and brown adipose tissue, which is responsible for the conversion of chemical energy into heat. Excessive accumulation of white adipose tissue and loss or dysfunction of brown adipose tissue will lead to a range of obesity-related metabolic disorders (23). Interestingly, although obesity is often associated with metabolic disorders and increased risk of chronic disease, some obese individuals maintain normal blood glucose, lipid, and blood pressure levels (no significant metabolic abnormalities). These individuals are therefore referred to as having MHO (10, 24, 25).

Emerging evidence suggests that the association between obesity and cancer risk may be mediated by multiple molecular pathways (26–29). One of the key mechanisms involves insulin resistance, which often accompanies obesity (26). Insulin resistance leads to hyperinsulinemia, promoting the activation of insulin-like growth factor (IGF) signaling pathways, which stimulate cell proliferation and inhibit apoptosis—key processes in cancer development. Furthermore, obesity is characterized by chronic low-grade inflammation, particularly in visceral adipose tissue (27, 28). Pro-inflammatory cytokines such as tumor necrosis factor-alpha (TNF-α), interleukin-6 (IL-6), and C-reactive protein (CRP) are often elevated in obese individuals. These inflammatory markers can contribute to tumorigenesis by creating a pro-tumor environment, enhancing cell proliferation, and promoting angiogenesis (27). In addition, adipokine dysregulation plays a crucial role in the obesity-cancer link (29). Adipokines, including leptin and adiponectin, are secreted by adipose tissue and have been shown to modulate metabolic and immune responses (29, 30). While leptin, often upregulated in obesity, has pro-tumorigenic effects by enhancing angiogenesis and cell proliferation, adiponectin, which is reduced in obesity, exhibits anti-inflammatory and anti-tumor properties (30).

The prevalence of MUO and MHO in the Chinese population varies due to different definition criteria (31). One study reported that according to the National Cholesterol Education Program-ATP-III, Karelis, Wildman, Chinese Diabetes Society, and the HOMA index criteria, the prevalence of MHO ranged from 4.2–13.6%, and the prevalence of MUO ranged from 10.6–20.1% when defining obesity based on BMI. The prevalence of MHO and MUO was significantly higher when the obesity was defined according to WC. In this study, the prevalence of MHO and MUO among participants with simple obesity was 6.86% and 12.18%, respectively, while the prevalence of MHO and MUO among participants with abdominal obesity was 20.79% and 25.76%, respectively. In a previous study, those with only obesity and no metabolic health problems (e.g., diabetes, hypertension, insulin resistance, dyslipidemia, or inflammation) had better health and reduced mortality by 30–50% (32). However, a meta-analysis of eight longitudinal studies showed that MHO increased the risk of all-cause mortality in the long term (≥10 years) (33). This suggested that MHO may be an intermediate state between MUO and MHNW. Previous studies found that MUO increased the risk of obesity-related cancers (colorectal, endometrial, liver, renal cell, gallbladder, and pancreatic) compared to MHNW, and MHO has also shown a relatively higher risk of obesity-related cancers. Another study investigated the association between the MHO and the risk of lung cancer and showed that obesity was a protective factor, especially in metabolically healthy people (33). The above findings suggested that there may be differences in the association between metabolic health status combined with obesity categories and the risk of various cancer types, and that different definitions of MHO may lead to different outcomes.

In addition, the metabolic risks associated with obesity are primarily related to the distribution of fat, but BMI does not fully and accurately reflect fat distribution and body composition (34, 35). Visceral adipose tissue, compared to subcutaneous adipose tissue, is strongly associated with obesity-related complications, including type 2 diabetes, non-alcoholic fatty liver disease, cardiovascular disease, and certain types of cancer (36). In fact, many studies reported that WC is a more accurate indicator that not only distinguishes overall obesity and abdominal obesity within the same BMI range, but also effectively reflects visceral fat (36). Recent studies focused on the association between the risk of cancer and MHO defined based on BMI criteria, while WC may be a better indicator for obesity assessment.

The strength of this study is that it is based on a large population. Nevertheless, some limitations remain. First, there are multiple definitions of MHO, so we used the definition criteria with the middle prevalence reported previously. Second, MHO may be a transitional intermediate stage, and the association between its transformation and cancer morbidity still warrants further study. Finally, the association between MHO and the risk of different cancers may be highly variable, so the association still needs to be further investigated by refining the tumor types.

## 5 Conclusions

In conclusion, both abdominal obesity and metabolically unhealthy status contribute to cancer morbidity and increase the risk of all-cause mortality. In contrast, simple overweight and obesity are protective factors against the risk of cancer and all-cause mortality in participants with MUO. This suggests that individuals should focus on weight management to maintain a metabolically healthy state, and should actively reduce the metabolism-related risk factors increased by abdominal obesity.

## Data Availability

The data analyzed in this study is subject to the following licenses/restrictions: Data and programming code is available upon request. Further enquiries can be directed to the corresponding author. Requests to access these datasets should be directed to xinzheng0405@163.com.
