# Supplementary material for: Metabolic obesity phenotypes and the risk of cancer: a prospective study of the Kailuan cohort
Source: Front Endocrinol (Lausanne). 2024 Oct 16;15:1333488. doi: 10.3389/fendo.2024.1333488 (PMC11521940; doi:10.3389/fendo.2024.1333488)
Supplement: Supplementary file 1 [file DataSheet1.docx]

**Supplementary Table 1. The Association between metabolic healthy obesity, overweight and normal weight grouped by BMI and cancer morbidity**

| **Type** | **Cancer/total participants** | **Model 1** | **p** | **Model 2** | **p** | **Model 3** | **p** |
| --- | --- | --- | --- | --- | --- | --- | --- |
| **MHNW** | 1355/23988 | ref. | ref. | ref. | ref. | ref. | ref. |
| **MUNW** | 756/11211 | 1.22 (1.11,1.33) | <0.001 | 1.15 (1.06,1.26) | 0.002 | 1.15 (1.05,1.26) | 0.002 |
| **MHOW** | 1052/19769 | 0.94 (0.86,1.02) | 0.113 | 0.94 (0.86,1.02) | 0.116 | 0.91 (0.84,0.99) | 0.031 |
| **MUOW** | 1183/19382 | 1.10 (1.01,1.19) | 0.021 | 1.05 (0.97,1.14) | 0.219 | 1.01 (0.93,1.10) | 0.823 |
| **MHO** | 378/6297 | 1.07 (0.95,1.20) | 0.266 | 1.08 (0.96,1.21) | 0.190 | 1.01 (0.90,1.14) | 0.864 |
| **MUO** | 679/11187 | 1.10 (1.01,1.21) | 0.044 | 1.07 (0.98,1.17) | 0.148 | 0.99 (0.90,1.10) | 0.922 |
| **P for trend** |  |  | 0.127 |  | 0.270 |  | 0.465 |

**Notes:** Model 1: adjusted for age and sex; Model 2: adjusted for Model 1, education, work, income, sedentary, physical activity, smoke, salt, drink, tea; Model 3, adjusted for Model2, hepatitis B, liver cirrhosis, gallstones, biliary polyps, fatty liver and creatinine.

**Abbreviation:** MHNW, metabolic healthy normal weight; MUNW, metabolic unhealthy normal weight; MHOW, metabolic healthy overweight; MUOW, metabolic unhealthy overweight; MHO, metabolic healthy obesity; MUO, metabolic unhealthy obesity; BMI, body mass index.

**Supplementary Table 2. The Association between metabolic healthy obesity, overweight and normal weight grouped by WC and cancer morbidity**

| **Type** | **Cancer/total participants** | **Model 1** | **p** | **Model 2** | **p** | **Model 3** | **p** |
| --- | --- | --- | --- | --- | --- | --- | --- |
| MHNW | 1595/30960 | ref. | ref. | ref. | ref. | ref. | ref. |
| MUNW | 1067/18124 | 1.16 (1.07,1.25) | <0.001 | 1.11 (1.02,1.19) | 0.012 | 1.11 (1.02,1.20) | 0.012 |
| MHO | 1190/19094 | 1.22 (1.13,1.32) | <0.001 | 1.19 (1.10,1.28) | <0.001 | 1.16 (1.08,1.26) | <0.001 |
| MUO | 1551/23656 | 1.31 (1.22,1.41) | <0.001 | 1.24 (1.15,1.33) | <0.001 | 1.21 (1.12,1.30) | <0.001 |
| **P for trend** |  |  | <0.001 |  | <0.001 |  | <0.001 |

**Notes:** Model 1: adjusted for age and sex; Model 2: adjusted for Model 1, education, work, income, sedentary, physical activity, smoke, salt, drink, tea; Model 3, adjusted for Model2, hepatitis B, liver cirrhosis, gallstones, biliary polyps, fatty liver and creatinine.

**Abbreviation:** MHNW, metabolic healthy normal weight; MUNW, metabolic unhealthy normal weight; MHO, metabolic healthy obesity (central); MUO, metabolic unhealthy obesity (central); WC, waist circumference.

**Supplementary Table 3. The Association between metabolic healthy score and all-cause mortality**

| **Group** | **Type** | **Cancer/total participants** | **Model 1** | **p** | **Model 2** | **p** | **Model 3** | **p** |
| --- | --- | --- | --- | --- | --- | --- | --- | --- |
| **Metabolic**  **type** | **Metabolic healthy status** | 5783/50054 | ref. | ref. | ref. | ref. | ref. | ref. |
|  | **Metabolic unhealthy status** | 7698/41780 | 1.54 (1.48,1.59) | <0.001 | 1.47 (1.42,1.52) | <0.001 | 1.44 (1.39,1.49) | <0.001 |
| **Score** | **Score0** | 1331/18780 | ref. | ref. | ref. | ref. | ref. | ref. |
|  | **Score1** | 4452/31274 | 1.68 (1.50,81.79) | <0.001 | 1.58 (1.48,1.68) | <0.001 | 1.56 (1.47,1.66) | <0.001 |
|  | **Score2** | 4768/27553 | 2.08 (1.96,2.21) | <0.001 | 1.93 (1.81,2.05) | <0.001 | 1.89 (1.78,2.01) | <0.001 |
|  | **Score3** | 2644/12898 | 2.51 (2.35,2.68) | <0.001 | 2.29 (2.14,2.45) | <0.001 | 2.23 (2.08,2.38) | <0.001 |
|  | **Score4** | 286/1329 | 2.91 (2.56,3.31) | <0.001 | 2.49 (2.19,2.84) | <0.001 | 2.44 (2.14,2.77) | <0.001 |
| **P for trend** | |  |  | <0.001 |  | <0.001 |  | <0.001 |

**Notes:** Model 1: adjusted for age and sex; Model 2: adjusted for Model 1, education, work, income, sedentary, physical activity, smoke, salt, drink, tea; Model 3, adjusted for Model2, hepatitis B, liver cirrhosis, gallstones, biliary polyps, fatty liver and creatinine.

**Supplementary Figure 1. The Association between Metabolic Healthy Status Combined with Obesity Categories and the Risk of Different Cancers**


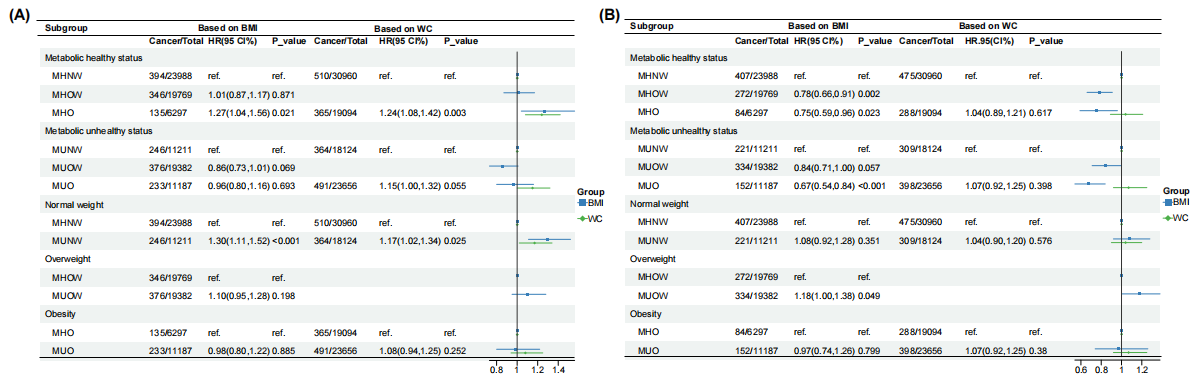


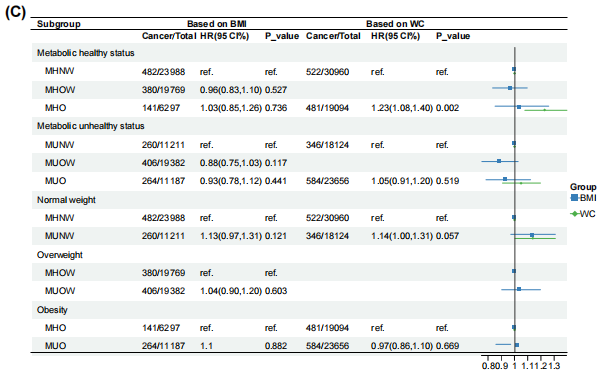


**Notes:** (A) cancer of the digestive system; (B) cancer of the respiratory system; (C) cancer of the other system

**Abbreviation:** MHNW, metabolic healthy normal weight; MUNW, metabolic unhealthy normal weight; MHOW, metabolic healthy overweight; MUOW, metabolic unhealthy overweight; MHO, metabolic healthy obesity; MUO, metabolic unhealthy obesity; BMI, body mass index; WC, waist circumference; HR, hazard ratio; CI, confidence interval.

**Supplementary Figure 2. The Association between Metabolic Healthy Status Combined with Obesity Categories and the Risk of All-cause Mortality**


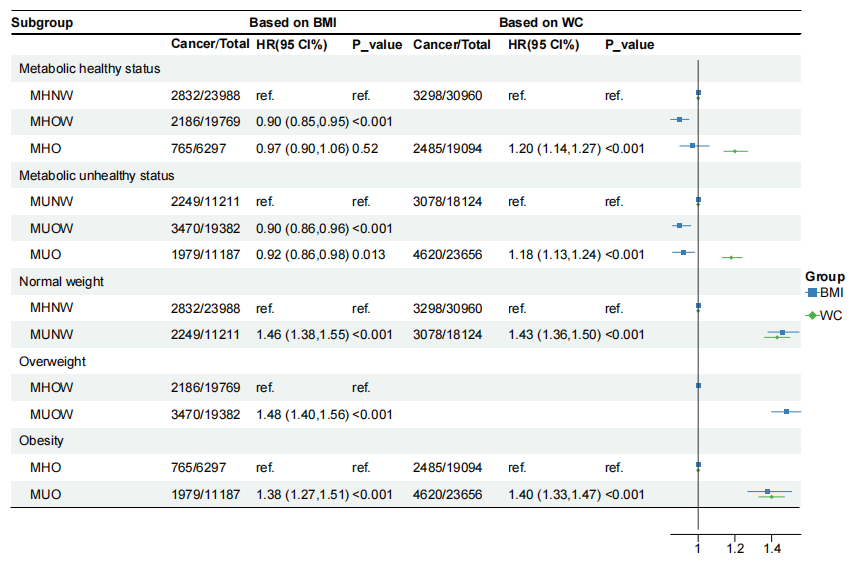


**Abbreviation:** MHNW, metabolic healthy normal weight; MUNW, metabolic unhealthy normal weight; MHOW, metabolic healthy overweight; MUOW, metabolic unhealthy overweight; MHO, metabolic healthy obesity; MUO, metabolic unhealthy obesity; BMI, body mass index; WC, waist circumference; HR, hazard ratio; CI, confidence interval.

**Supplementary Figure 3. The Association between Metabolic Healthy Status Combined with Obesity Categories and the Risk of Cancer after Exclusion of Participants with Cancers Occurring within One Year and Additional Adjustment for LDL and TC**


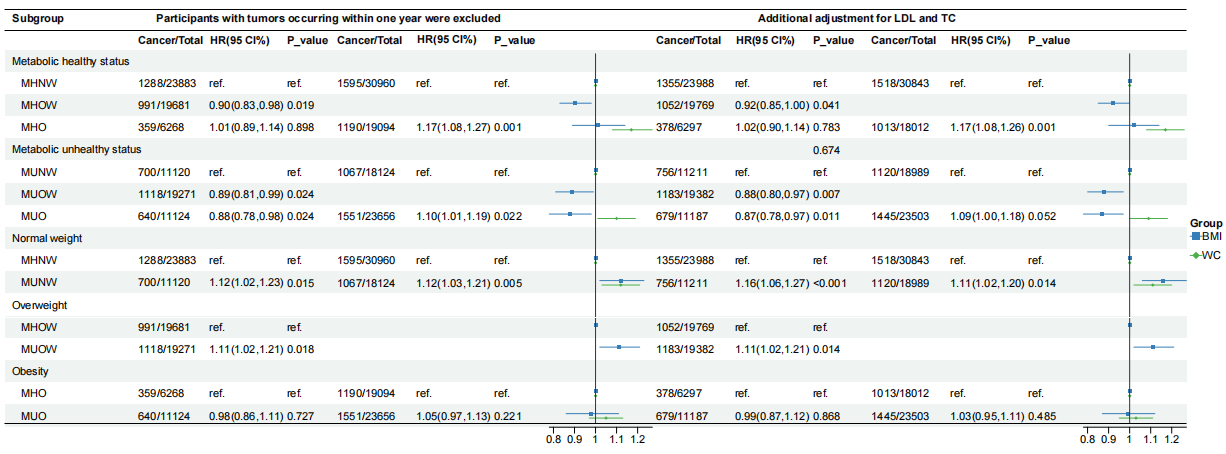


**Abbreviation:** MHNW, metabolic healthy normal weight; MUNW, metabolic unhealthy normal weight; MHOW, metabolic healthy overweight; MUOW, metabolic unhealthy overweight; MHO, metabolic healthy obesity; MUO, metabolic unhealthy obesity; BMI, body mass index; WC, waist circumference; HR, hazard ratio; CI, confidence interval.
